# Time-course proteomics dataset to monitor protein-bound methionine oxidation *in Bacillus cereus* ATCC 14579

**DOI:** 10.1016/j.dib.2018.03.030

**Published:** 2018-03-10

**Authors:** Jean-Paul Madeira, Béatrice Alpha-Bazin, Jean Armengaud, Catherine Duport

**Affiliations:** a SQPOV, UMR0408, Avignon Université, INRA, F-84914 Avignon, France; b CEA, DRF/Joliot/DMTS/SPI, Li2D, Laboratory "Innovative technologies for Detection and Diagnostics", Bagnols-sur-Cèze F-30200, France

**Keywords:** Methionine oxidation, Cellular proteome, Exoproteome, Methionine sulfoxide reductase, *Bacillus cereus*

## Abstract

Aerobic respiratory growth generates endogenous reactive oxygen species (ROS). ROS oxidize protein-bound methionine residues into methionine sulfoxide. Methionine sulfoxide reductases catalyze the reduction of methionine sulfoxide to methionine in proteins. Here, we use high-throughput nanoLC-MS/MS methodology to establish detailed maps of oxidized proteins from *Bacillus cereus* ATCC 14579 ΔpBClin15 and its mutant for which the methionine sulfoxide reductase AB gene (*msrAB*) has been inactivated (Madeira et al., 2017) [1]. Lists of oxidized peptides and proteins identified at early exponential, late exponential and stationary growth phases are supplied in this article as data files. Raw data are deposited at the ProteomeXchange Consortium via the PRIDE partner repository with the dataset identifiers, PXD006169 and PDX006205 (http://www.ebi.ac/uk). Given the importance of methionine oxidation in several key cellular processes and its impact in the field of medical and food microbiology, this paper should be useful for further insightful redox studies in *B. cereus* and its numerous relatives.

**Specifications Table**

**Table:** 

**Subject area**	Microbiology
**More specific subject area**	Proteomics
**Type of data**	Figure, Tables.
**How data was acquired**	Protein extracts from biological triplicates were subjected to SDS-PAGE and proteolyzed in-gel. Peptides were analyzed using an LTQ-Orbitrap XL hybrid mass spectrometer (ThermoFisher) coupled to an Ultimate 3000 nRSLC system (Dionex, ThermoFisher).
**Data format**	Analyzed
**Experimental factors**	Wild-type and Δ*msrAB Bacillus cereus* strains were grown under aerobiosis in defined MOD medium supplemented with glucose as the carbon source [5].
**Experimental features**	Cellular proteins were prepared from cell lysates obtained with a Precellys 24 disruptor (Bertin Technologies). Exoproteins were obtained by trichloroacetic acid precipitation of the filtered culture supernatant. Cellular proteins and exoproteins were collected at the early exponential, late exponential and stationary growth phases.
**Data source location**	France
**Data accessibility**	Analyzed datasets are within this article and raw data are available via the PRIDE partner repository (http://www.ebi.ac.uk/pride) with the dataset identifiers, PRIDE: PXD006169 (cellular proteome) and PRIDE: PXD006205 (exoproteome).

**Value of the data**

• This is the first dataset that provides a whole-cell global view of protein-bound methionine oxidation in *B. cereus*.
• The identified methionine sulfoxide peptides contribute to establish a map of oxidized proteins in this pathogen.
• This dataset allows visualizing the cellular and extracellular impact of methionine sulfoxide reductase MsrAB.
• This dataset should be useful in prioritizing proteins for further studies to determine their potential as redox regulator of virulence.

## 1 Data

**Fig. 1:**
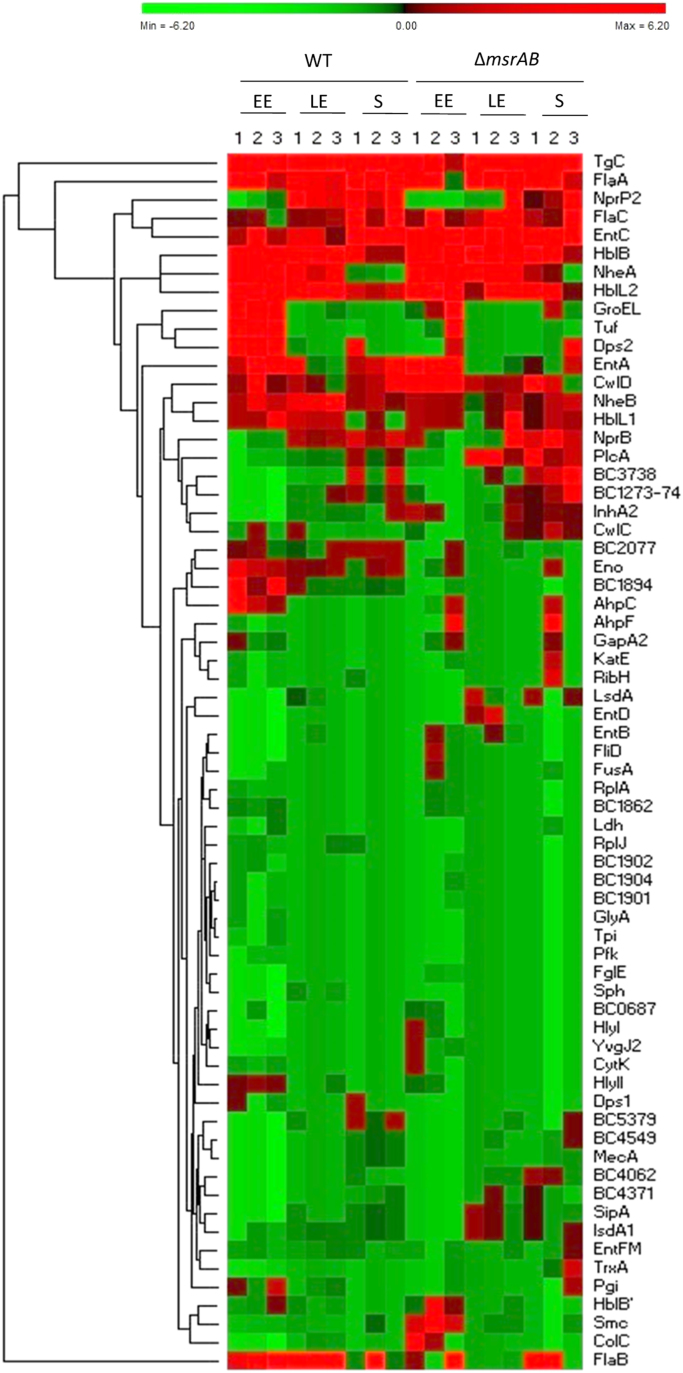
Heatmap display of hierarchical clustering of the oxidized protein abundance (normalized by *z*-score) in filtered supernatant of *B. cereus*. Each column represents one of the three biological replicates harvested at early exponential (EE, OD_600_ = 0.15), late exponential (LE, OD_600_ = 2.4) and stationary (S, OD_600_ = 3.9) growth phases for the wild-type strain (WT) and the mutant strain (Δ*msrAB*). In each sample, proteins with high levels of oxidation are indicated in red and proteins with low levels of oxidation are indicated in green. A dendrogram is displayed at the left of the heatmap to visualize the Euclidean correlation between each pair of protein. The rows of heatmap represent the 70 oxidized exoproteins (Supplementary Table S4).

This proteomics dataset comprises the whole set of peptides and Met(O) peptides identified in the cellular proteome (Supplementary Table 1) and exoproteome (Supplementary Table 2) of *Bacillus cereus* ATCC 14579 ΔpBClin15 (Wild-type, [2]) and its mutant *ΔmsrAB* [1]. A total of 8720 and 3417 Met(O)peptides were detected in the cellular proteomes and exoproteomes, respectively. Supplementary Tables 3 and 4 show the 174 identified oxidized cellular proteins that were classified in 19 functional classes and the 70 detected oxidized exoproteins that were classified in 14 functional classes. From Supplementary Tables 3 and 4, we generated heatmaps to visualize clustered oxidized proteins in cellular (Supplementary Fig. 1) and exoproteome (Fig. 1), according to the growth phase. Global changes between the wild-type and *ΔmsrAB* mutant strains can also been observed. Fig. 1 shows that exoproteins with the highest oxidation levels are mainly virulence factors. Raw LC–MS/MS files are available via the PRIDE [3] partner repository with the dataset identifier PXD006169 (cellular proteome) and PXD00006205 (exoproteome).

## 2 Experimental design, materials and methods

### 2.1 *B. cereus* cultures

*B. cereus* wild-type and Δ*msrAB* cells were grown in a 3-l glass BioFlo/CelliGen 115 (Eppendorf, New Brunswick Scientific, Juelich, Germany) bioreactor with a working volume of 2 l, as described previously [2]. Three independent biological replicates were cultured in MOD medium [4] supplemented with glucose as carbon source under aerobiosis (pO_2_ =100%). Cell pellets and culture supernatants were harvested for each biological replicate at early exponential (EE, OD_600_ = 0.15), late exponential (LE, OD_600_ = 2.4) and stationary (S, OD_600_ = 3.9) growth phases.

### 2.2 Peptide preparation

Cell pellets and supernatants were separated by centrifugation (10,000*g*, 10 min, 4 °C). Exoproteins were isolated from filtered supernatants by TCA precipitation and soluble cellular proteins were extracted from cell lysates as described previously [1], [2], [5], [6]. To avoid any artefactual oxidation, exoproteins and cellular proteins were resolved on denaturing NuPAGE 4–12% Bis-Tris gels (Invitrogen, ThermoFisher, Courtaboeuf, France) during a 5 min electrophoretic migration using NuPAGE antioxidant added to the NuPAGE running buffer. Proteins were then directly subjected to in-gel proteolysis using sequencing grade trypsin (Promega, Charbonnières les Bains, France) and ProteaseMAX surfactant (Promega) at 0.01%, and the resulting peptides were immediately subjected to nanoLC-MS/MS identification. Chromatographic separation of tryptic peptides was done with a Dionex nanoscale C18 capillary column using a 90-min gradient (extracellular peptide digests), or a 180-min gradient (cellular peptide digests), from 4% to 40% solvent B (0.01% HCOOH, 100% CH_3_CN) with solvent A being 0.01% HCOOH, 100% H_2_O [7], [8].

### 2.3 LC–MS/MS and data analysis

Peptide samples were analyzed on LTQ-Orbitrap XL hybrid mass spectrometer (ThermoFisher) coupled to an Ultimate 3000 nRSLC system (Dionex, ThermoFisher) as reported previously [9]. Mass spectrometry analysis was carried out with full scans (300- to 1800 *m/z*) in data dependent mode using a TOP3 strategy consisting in a full MS scan followed by MS/MS analysis of the three most abundant peptide signals. MS/MS data obtained from all LC–MS analyses were searched against an in-house polypeptide sequence database using MASCOT (version 2.3.02) search engine from Matrix Science. The parameters used for data analysis included trypsin as the protease with a maximum of two missed cleavages. Carboxyamidomethylation of cysteine (+57.0215) was specified as a fixed modification and oxidation of methionine (+15.9949) as a variable modification. Mass tolerances were set at 5 ppm for precursor ions and 0.5 Da for fragment ions. Peptides were considered as identified with a peptidic identity threshold below a *p*-value of 0.05 [10]. Proteins were validated when at least two different peptides were detected. False-positive identification of proteins was estimated using the corresponding reverse decoy database as below 1% with these parameters. For comparative analysis only the proteins containing at least two oxidized peptides in two replicates of the same sample were considered as oxidized. Hierarchical clustering of oxidized proteins was carried out with PermutMatrix Software [11].
