# Supplementary material for: Time-course proteomics dataset to monitor protein-bound methionine oxidation in Bacillus cereus ATCC 14579
Source: Data Brief. 2018 Mar 10;18:394–8. doi: 10.1016/j.dib.2018.03.030 (PMC5996235; doi:10.1016/j.dib.2018.03.030)
Supplement: Supplementary file 2 — Supplementary material [file mmc1.pptx]

## Slide 1
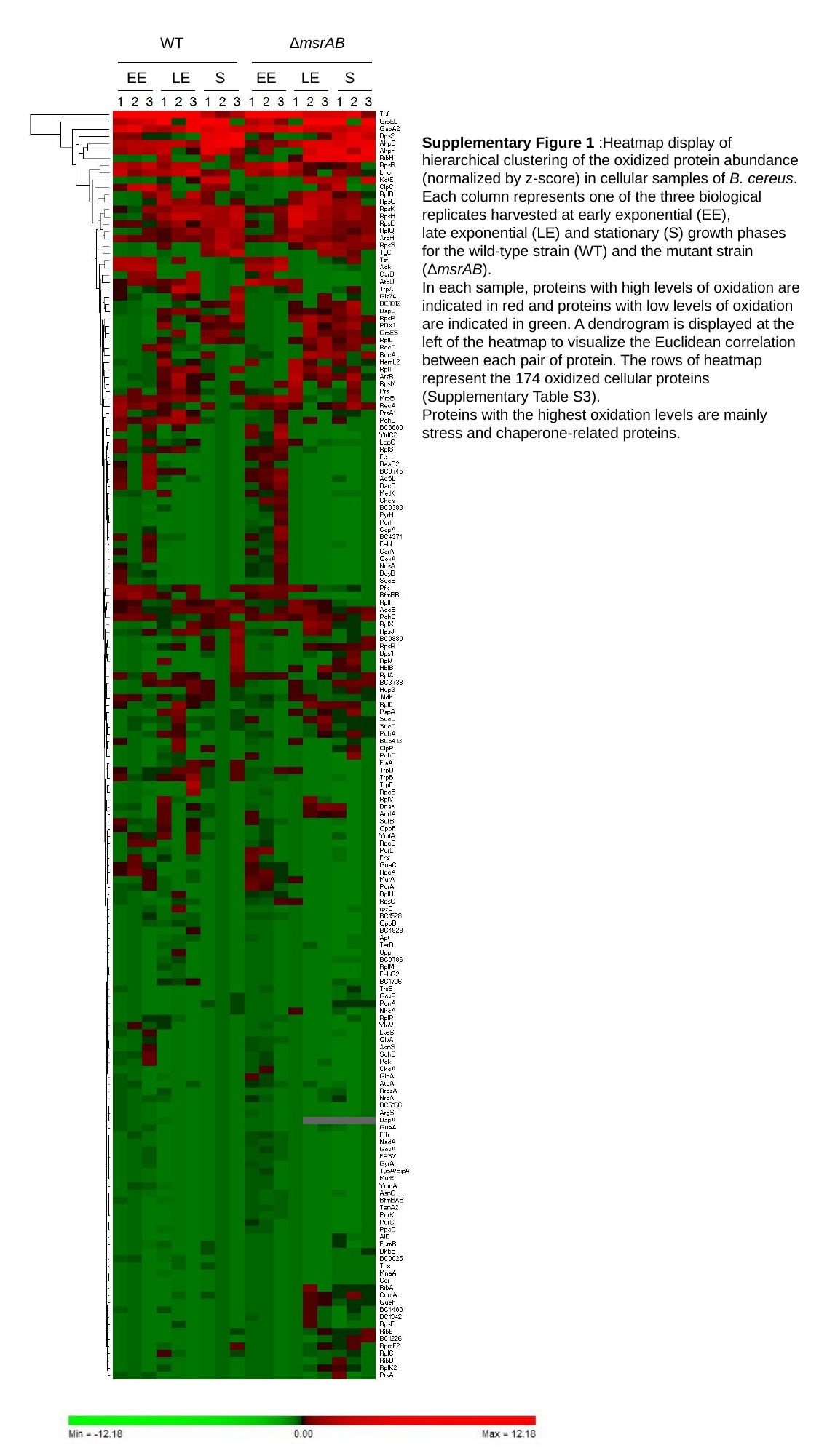

WT
ΔmsrAB
EE LE S
EE LE S
Supplementary Figure 1 :Heatmap display of
hierarchical clustering of the oxidized protein abundance
(normalized by z-score) in cellular samples of B. cereus.
Each column represents one of the three biological
replicates harvested at early exponential (EE),
late exponential (LE) and stationary (S) growth phases
for the wild-type strain (WT) and the mutant strain
(ΔmsrAB).
In each sample, proteins with high levels of oxidation are
indicated in red and proteins with low levels of oxidation
are indicated in green. A dendrogram is displayed at the
left of the heatmap to visualize the Euclidean correlation
between each pair of protein. The rows of heatmap
represent the 174 oxidized cellular proteins
(Supplementary Table S3).
Proteins with the highest oxidation levels are mainly
stress and chaperone-related proteins.
